# Broadband wearable acoustic sensing for continuous cardiopulmonary monitoring

**DOI:** 10.1038/s41378-026-01460-5

**Published:** 2026-09-29

**Authors:** Pengfan Wu, Tao Liu, Dongxiao Li, Hanjie Dou, Jiaqian Yang, Hongliang Wang, Hengyu Guo, Xiaojing Mu

**Affiliations:** 1 Key Laboratory of Optoelectronic Technology & Systems Ministry of Education, International R & D center of Micro-nano Systems and New Materials Technology, Chongqing University, Chongqing, 400044 P.R. China; 2 Jialingjiang Laboratory, Chongqing, 401331 China; 3 Science and Technology on Electronic Test and Measurement Laboratory, State key Laboratory of Extreme Environment Optoelectronic Dynamic Measurement Technology and Instrument, North University of China, Taiyuan, 030051 P.R. China; 4 College of Physics, Chongqing University, Chongqing, 400044 China

**Keywords:** Sensors, Electrical and electronic engineering

## Abstract

The human body continuously generates weak, broadband acoustic–mechanical signals that encode rich cardiopulmonary information, yet their reliable capture in real-world settings remains constrained by motion artifacts, limited bandwidth, and suboptimal mechanical coupling. Here, we present a wearable, wireless stethoscope that enables high-fidelity acquisition of heart and respiratory sounds through a mechanically compliant, broadband sensing interface. The system is built on a piezoelectric micromachined ultrasonic transducer (PMUT) engineered to achieve ultrahigh sensitivity (–167.5 dB), a wide operating bandwidth (10 Hz–10 kHz), and a flat frequency response (±0.5 dB), enabling accurate reconstruction of subtle physiological signals across a broad spectral range. Integration with soft, skin-conformal packaging and a robust wireless readout minimizes motion-induced noise and supports stable, long-term monitoring during daily activities. Validation across multiple participants demonstrates strong agreement with clinical-grade systems, including consistent auscultation at standard cardiac sites and accurate heart-rate tracking during dynamic exercise. Furthermore, coupling the sensing platform with a residual neural network enables automated classification of five respiratory states (awake, asleep, apnea, rhonchi, and wheeze) with an accuracy of 98.7%. This work establishes a scalable, low-cost framework for continuous, high-fidelity cardiopulmonary monitoring in real-world environments.

## Introduction

Continuous monitoring of cardiopulmonary function is essential for the early detection and management of major diseases such as cardiovascular disease (CVD)^1^ and obstructive sleep apnea (OSA)^2–4^, which together impose a substantial global health burdens^5–7^. Despite significant advances in modern medicine, many cardiopulmonary conditions remain underdiagnosed due to the lack of accessible and continuous monitoring tools^8,9^. Current clinical approaches predominantly rely on episodic measurements using hospital-based imaging modalities, including echocardiography, magnetic resonance imaging, and computed tomography^10–17^. While these techniques provide detailed structural and functional information, they are costly, resource-intensive, and impractical for long-term or real-world monitoring. Consequently, there is a critical need for wearable, non-invasive technologies capable of continuously capturing physiological signals with high fidelity in daily life^18^.

The human body continuously generates weak, broadband acoustic–mechanical signals that encode rich physiological and pathological information^19^. These signals originate from fundamental biomechanical processes, including cardiac valve motion, blood flow–induced vibrations, and airflow dynamics within the respiratory tract. As these mechanical waves propagate through biological tissues, they carry organ-specific signatures that reflect both normal physiological function and pathological alterations. Subtle variations in these signals can provide early indicators of disease progression, often preceding clinically observable symptoms. For example, abnormal heart sounds may reveal structural or hemodynamic abnormalities in the heart^20,21^, whereas changes in respiratory sounds may indicate airway obstruction, fluid accumulation, or inflammation^22–25^. As a non-invasive and cost-effective diagnostic modality, auscultation enables the detection of subtle physiological changes that are otherwise imperceptible, offering a unique pathway for early disease identification.

Although traditional auscultation has long been employed in clinical practice, it largely relies on the clinician’s experience and auditory perception, leading to inherent subjectivity and limited reproducibility^26^. The transition to digital stethoscopes has enabled the conversion of acoustic signals into electrical signals for amplification, filtering, and quantitative analysis, thereby improving diagnostic consistency^27–33^. Recent advances in wearable sensing technologies have further extended auscultation beyond clinical settings, enabling continuous monitoring and remote healthcare applications^34^. These systems utilize diverse sensing mechanisms, including piezoelectric transducers, microelectromechanical systems (MEMS) accelerometers, optical fiber sensors, and triboelectric nanogenerators, to detect physiological signals with improved sensitivity and functionality^35–39^. Despite these advancements, several fundamental challenges remain unresolved. First, many existing systems are based on rigid or bulky device architectures that limit mechanical conformity to the skin, leading to poor acoustic coupling and reduced signal fidelity during motion. Second, the inherently broadband nature of physiological acoustic signals, spanning from infrasonic components to several kilohertz, is not fully captured by sensors with limited bandwidth or non-uniform frequency responses. This results in the loss of diagnostically relevant spectral features. Third, motion-induced artifacts and environmental noise significantly degrade the signal-to-noise ratio under real-world conditions, particularly during physical activity or long-term monitoring. Fourth, although digital signal processing and machine learning techniques have been introduced, many systems lack seamless integration between high-quality signal acquisition and intelligent data analysis, hindering automated and continuous diagnosis. Addressing these limitations requires a sensing platform that simultaneously achieves high sensitivity, broadband response, mechanical compliance, and robust system-level integration.

Here, we report a fully integrated, wireless, and flexible stethoscope for continuous mechanoacoustic sensing of cardiopulmonary activity. The system is built on a piezoelectric micromachined ultrasonic transducer (PMUT) that is engineered to achieve ultrahigh sensitivity, a wide operating bandwidth, and an exceptionally flat frequency response, enabling accurate reconstruction of weak and broadband physiological signals. To enable stable operation in dynamic environments, the sensing unit is integrated with soft, skin-conformal packaging and flexible electronics that improve mechanical coupling with the body while minimizing motion-induced artifacts. The wireless architecture further allows real-time data transmission and user-friendly operation, supporting continuous monitoring during daily activities without restricting mobility. We validate the system through human studies, demonstrating reliable acquisition of heart and respiratory sounds across multiple auscultation sites and strong agreement with clinical reference systems. Furthermore, the system enables robust heart rate tracking during dynamic physical activities, highlighting its resilience to motion and environmental disturbances. By integrating deep learning algorithms with the sensing platform, we further achieve automated classification of multiple respiratory states, enabling intelligent interpretation of physiological signals for applications such as sleep monitoring and disease screening. This work establishes a scalable and low-cost framework for continuous, high-fidelity cardiopulmonary monitoring, advancing wearable acoustic sensing toward autonomous and real-world healthcare applications.

## Results and discussion

### Design and structure of a wearable auscultation system

**Fig. 1:**
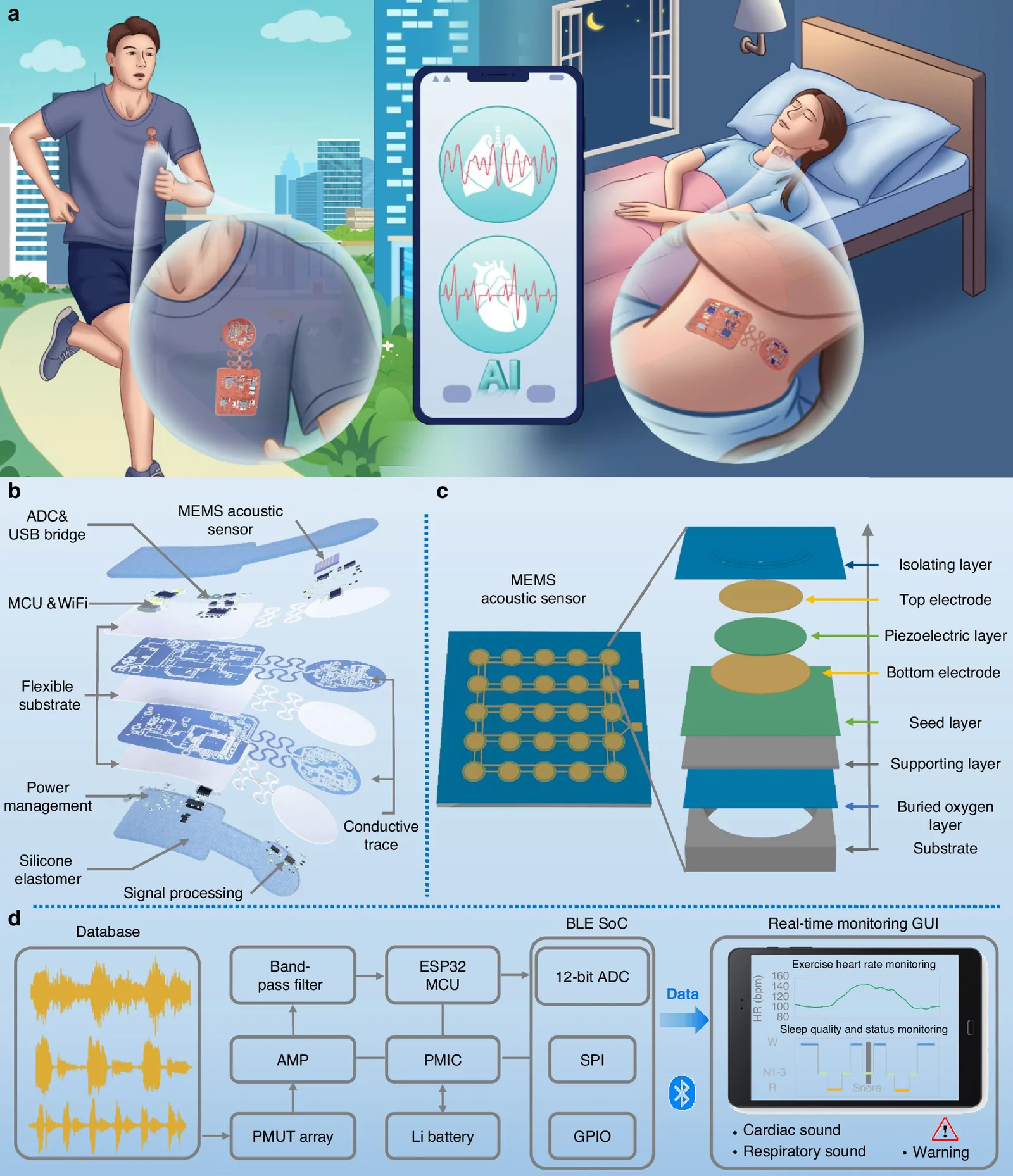
Integrated system-level wearable stethoscope for cardiorespiratory health monitoring. **a** Images of a wireless monitoring system for movement and sleep, for real-time recording of cardiopulmonary sound signals, and for deep learning-based status classification and disease monitoring using a mobile terminal. **b** Exploded view schematic illustration of the wearable stethoscope. **c** Structural scheme of the MEMS acoustic device. **d** Schematic diagram of the process of automatic and objective diagnosis of physiological status through deep learning in a wearable stethoscope

The key functions and design of the fully integrated wireless wearable stethoscope are illustrated in Fig. 1. By combining MEMS processing technology, flexible substrates, system integration, and flexible packaging, the device offers a portable wearable auscultation system with a high signal-to-noise ratio for cardiopulmonary monitoring. As shown in Fig. 1a, the stethoscope functions as a wearable system that continuously collects cardiopulmonary data related to exercise and sleep. The data were wirelessly transmitted to a terminal for real-time status monitoring and disease diagnosis. The stethoscope’s compact size and soft mechanical properties allow it to form a close fit with the skin on the chest and respiratory surface, ensuring a comfortable experience, even during continuous monitoring scenarios such as exercise and sleep. Additionally, the integration of machine learning-based diagnostic technology empowers users to monitor their physical condition anytime and anywhere, eliminating the need for dynamic coordination with healthcare providers.

Figure 1b provides a detailed exploded view of the densely packed multilayer structure of the flexible sensor. This structure includes a flexible printed circuit board (FPCB), a PMUT, electronic components, a rechargeable battery, a low-power Bluetooth module, and a flexible silicone encapsulation layer. The FPCB features a 100-μm-thick polyimide layer as the supporting substrate. The top and bottom surfaces of this substrate are patterned with 12-μm-thick rolled annealed copper (Cu). Each surface is further coated with a 50-μm- thick polyimide encapsulation layer to form an insulating layer. Various functional electronic components are then integrated onto the FPCB, enabling the sensor’s advanced capabilities (Fig. S1). Despite incorporating multiple rigid electronic components, the deformable FPCB substrate, serpentine interconnects, and flexible packaging materials provide the entire system with low-modulus elastic mechanics. The PMUT serves as the core sensing component for cardiopulmonary sound monitoring, and its multilayer stacked structure is illustrated in Fig. 1c. Compared to traditional microphones, such as dynamic or electret microphones, the PMUT is more compact (2 × 2 mm²), lighter (0.88 mg), and offers a broader frequency response range (10 Hz–10 kHz). Further details about the PMUT will be discussed in the next section. The bluetooth low energy (BLE) module (ESP32) facilitates high-speed wireless data transmission, while the entire sensing system is powered by a rechargeable 60 mAh lithium-ion polymer battery. To enhance skin compatibility and provide softer mechanical properties, a distributed layout scheme is employed. Specifically, MEMS sensing components and peripheral signal processing circuits are housed in the left circular region of the FPCB, while the battery, power management circuits, microprocessor, and wireless transmission module are positioned in the right area (see Fig. S2 for circuit details). This layout not only improves skin compatibility but also effectively minimizes circuit noise interference, enabling the sensor to achieve a high signal-to-noise ratio (SNR) of 40.09 dB (Fig. S3). A comprehensive comparison of the wearable stethoscope with recently reported stethoscopes and a commercial stethoscope (3 M Littmann CORE) is shown in Table S1. Different areas of the system are interconnected using serpentine traces. This serpentine interconnect structure enhances deformation flexibility, allowing the sensing system to adapt seamlessly to the skin’s contours and movements. As a result, both wearing comfort and sensor durability are significantly improved. The wearable stethoscope is secured to the skin using 3M 9907T biomedical adhesive. This flexible interface ensures a gentle attachment, avoiding irritation or discomfort, even during extended contact with sensitive skin areas.

Figure 1d illustrates the overall workflow of the stethoscope, encompassing signal acquisition, preprocessing, remote transmission, and deep learning. Initially, the MEMS acoustic transducer positioned on the chest or neck captures mechanical acoustic signals generated by cardiac and respiratory activities. These signals are then preprocessed using a filter amplification circuit and converted into digital signals by an analog-to-digital converter. The digital signals are transmitted in real time to a terminal device (such as a mobile phone, personal computer, or cloud server) via the BLE chip for further data processing. Next, a denoising algorithm removes redundant noise, while a neural network model classifies and interprets the signal data. A custom terminal application provides a graphical user interface for real-time recording, storage, and display of the relevant physiological signal data. By integrating micro-nano sensing, system integration, machine learning, and flexible packaging technologies, this wearable stethoscope system offers a promising solution for portable diagnostic tools, paving the way for advanced flexible and wearable healthcare devices.

### Device design and characterization

**Fig. 2:**
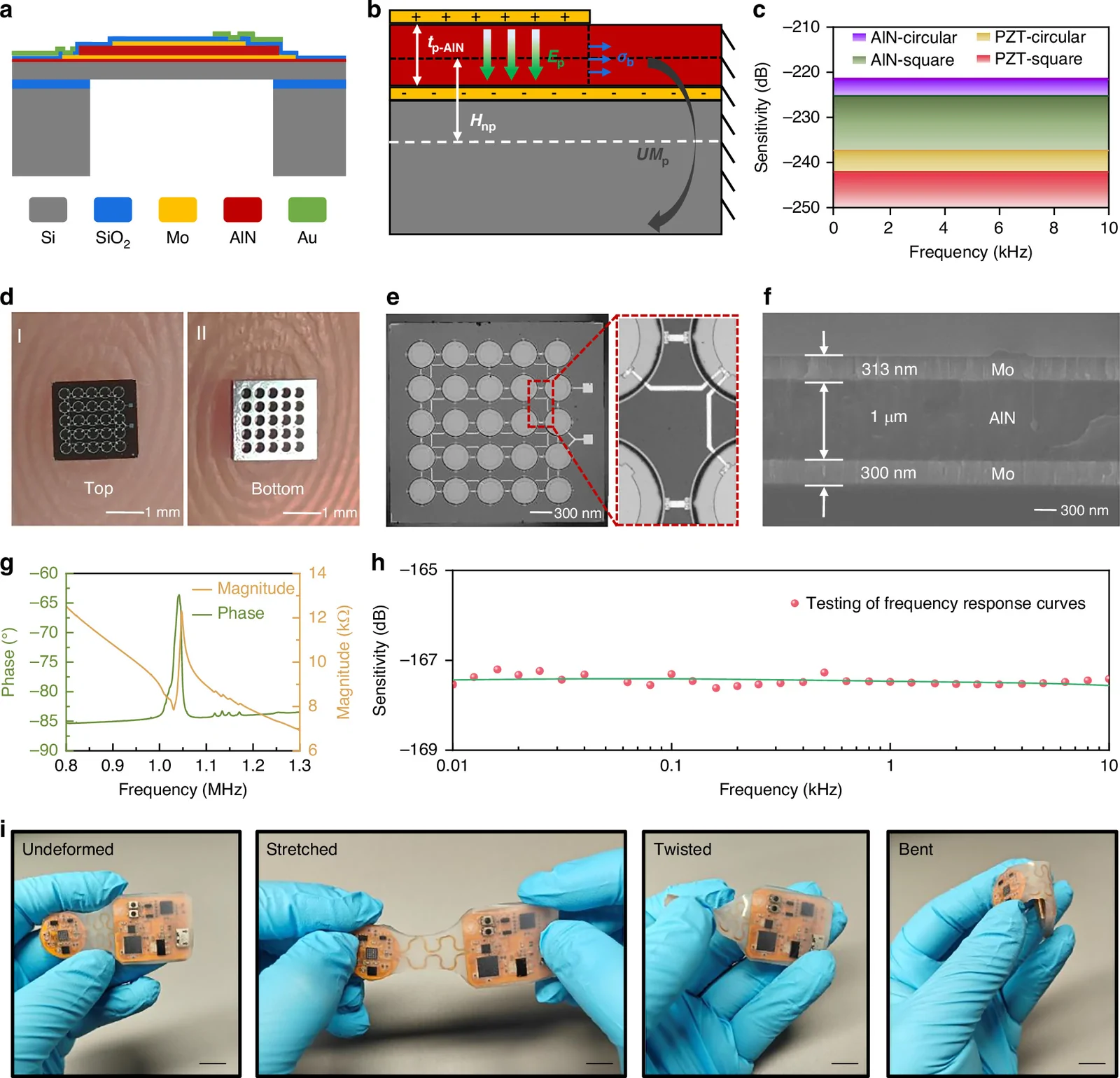
Design principle and characterization of the device. **a** Cross-sectional view of the three-dimensional structure of the acoustic sensor based on SOI wafer. **b** The 2D axisymmetric view of the vibration principle of PMUT piezoelectric films. **c** Finite element simulation comparison of relevant parameters of acoustic sensors of PZT, AlN and the corresponding two shapes. **d** Photos of the top (Ⅰ) and bottom (Ⅱ) of the MEMS acoustic chip. **e** Optical microscope image of the prepared acoustic chip and close-up details of a microelement. **f** SEM cross-sectional view of the Mo/AlN/Mo film structure of the prepared acoustic chip. **g** Resonant frequency response of the electrical impedance magnitude and phase of the MEMS chip in air. **h** Sensitivity test curve of the packaged MEMS sensor in the low-frequency wide-band range in water. **i** Image display of the flexible device under undeformed, stretched, twisted and bent. Scale bar: 1 mm

The PMUT serves as the receiving sensor in the wearable auscultation system, converting cardiopulmonary sound signals into electrical signals through the piezoelectric effect^40,41^. Figure 2a presents a schematic diagram of the structure of a single PMUT. The PMUT primarily consists of a top electrode, a piezoelectric film, a bottom electrode, and a cavity (Please refer to Table S2 for detailed material parameter settings). The cavity induces a bending moment along the neutral axis of the stress gradient within the film’s thickness direction, specifically in the area covered by the electrode. This bending moment generates an in-plane electromechanical strain in the piezoelectric film section beneath the top electrode, causing the film to vibrate (Detailed explanation of working principle refer to Fig. S4). Due to the d_31_ piezoelectric effect, an electric field is established between the top and bottom electrodes, as illustrated in Fig. 2b. Notably, the sensor achieves maximum conversion efficiency when the radius of the top electrode is 70% of the cavity radius (Fig. S5).

In physiological signal monitoring, PMUT requires high sensitivity, wide bandwidth, and good flatness. To develop high-performance sensing devices, we optimized the materials—lead zirconate titanate (PZT) and aluminum nitride (AlN)—and geometries—square and circular—of the PMUT using COMSOL multiphysics simulations (refer to Methods and Fig. S6 for further details). Considering the frequency impact of the package on the chip, we designed the PMUT’s resonant frequency to be 1 MHz to achieve flat sensitivity in the low-frequency range (Refer to Fig. S6 for the frequency response curves of the sensor’s full frequency band and local amplification). Figure 2c illustrates the receiving sensitivity of various PMUT designs within the 10 Hz–10 kHz frequency range, as determined by finite element simulations. Generally, for the same structural design and driving voltage, the receiving sensitivity of a PMUT is proportional to the ratio of the piezoelectric constant (d_31_) to the dielectric constant (ε_r_) of the material. Compared to PZT thin films, AlN exhibits a higher ratio (Table S3), which results in PMUT based on AlN demonstrating higher d_31_/ε_r_ receiving sensitivity. Next, we examine the impact of the PMUT structure on sensitivity. At the same resonant frequency, the circular film structure exhibits a more uniform stress distribution under pressure compared to the square film. This uniformity enables the circular film to produce a larger displacement under the same excitation, resulting in higher sensitivity^42^. Additionally, the mechanical impedance of the circular film is more consistent, allowing for better energy coupling with the surrounding medium and reducing energy loss. Due to the advantages in sensitivity (−221.5 dB), bandwidth (10 Hz–10 kHz), and flatness (±0.5 dB) offered by the AlN-based acoustic chip with a circular structure, this design was chosen for the sensor. To further enhance receiving sensitivity, we connected multiple PMUT units in parallel, allowing for greater charge accumulation during operation. The array design also increases the contact area with the skin, thereby capturing more cardiopulmonary-related acoustic signals. As a result, this design boosts the recognition capability and reliability of the auscultation system.

Next, we completed the fabrication of the PMUT using a standard MEMS process (see Methods and Fig. S7 for fabrication details). Figure 2d presents the top and back views of the PMUT, with the sensor array designed to occupy a compact 2 × 2 mm² area. Figure 2e includes an optical microscope image of the PMUT array, along with a magnified view of a single PMUT unit. A cross-sectional scanning electron microscope (SEM) image of the fabricated Mo/AlN/Mo stack is shown in Fig. 2f, clearly illustrating the distinct boundaries between the layers. The magnetron-sputtered AlN film demonstrates excellent crystal orientation. Mo was selected as the material for the top and bottom electrodes due to its superior lattice matching with AlN. Energy dispersive X-ray spectroscopy (EDS) was employed to analyze the element distribution across the cross-section (Fig. S8). The results reveal a uniform distribution of Al and N elements in the piezoelectric layer, while Mo elements are located on the upper and lower sides of the layer as electrodes. Each layer is tightly bonded and clearly distinguishable. X-ray diffraction (XRD) measurements of the AlN film reveal that the (002) peak of the AlN film appears around 36° (Fig. S9). The impedance analysis results align closely with the simulation data (Fig. S6a), confirming that the resonant frequency of the PMUT sensor is 1.04 MHz (Fig. 2g). This resonant frequency not only ensures high sensitivity (−167.5 dB) but also offers excellent flatness (±0.5 dB) across the working bandwidth (10 Hz–10 kHz) (Fig. 2h). For flexible packaging, Ecoflex-0030 silicone was used to enclose the densely packed electronic components. Figure 2i shows the packaged device under various mechanical deformations: from left to right, the device is shown undeformed, stretched, twisted, and bent. These results highlight the robust mechanical properties of the flexible stethoscope, which can adapt to the stability, low modulus, and elastic response required for wearable applications.

### The auscultation system monitors the performance of cardiac and respiratory sounds

Heart sounds are mechanical wave phenomena generated by myocardial contraction, the closure of heart valves, and the impact of blood against the ventricular and arterial walls. During a cardiac cycle, the myocardium produces four distinct sounds. The first heart sound (S1) occurs during the early phase of cardiac contraction, resulting from the closure of the mitral and tricuspid valves. It has a deep and continuous tone. The second heart sound (S2) occurs at the onset of cardiac diastole, caused by the closure of the aortic and pulmonary valves, and is characterized by a higher frequency but shorter duration. Among these, the frequencies of S1 and S2 typically range between 10 Hz and 500 Hz.

**Fig. 3:**
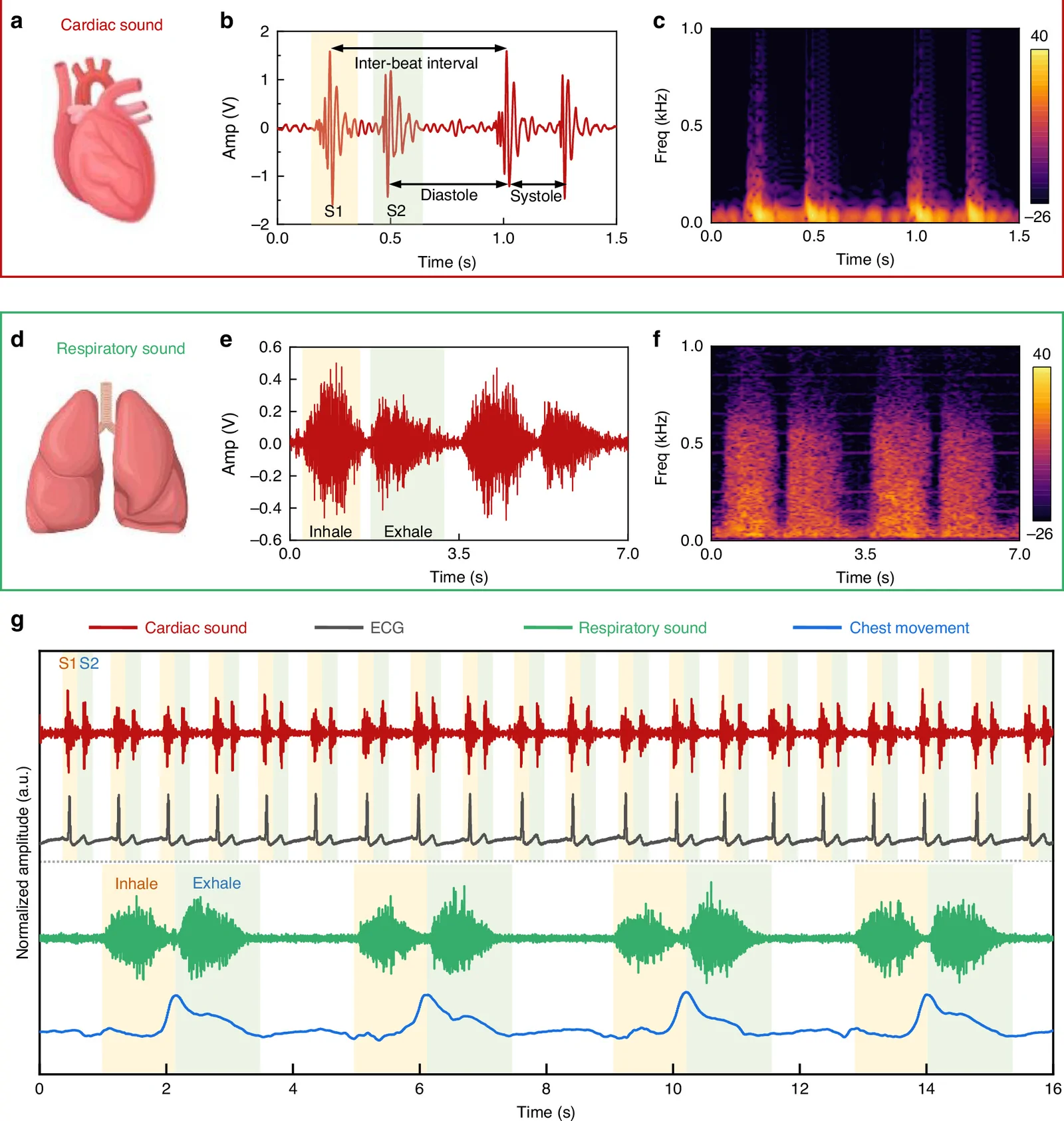
The auscultation system monitors the performance of cardiac and respiratory sounds. **a** Schematic diagram of the human heart. **b** Time-series plot of the 1.5-s window Cardiac sounds, measured from a healthy subject. **c** Spectrogram of the time-series plot showing the frequency range of the measured signals. **d** Schematic diagram of the human lungs. **e** Time-series plot of the 7-s window Respiratory sounds from a healthy subject with normal breathing. **f** Spectrogram of the time-series plot showing the frequency range of the lung signals. **g** Commercial electrocardiogram devices and chest exercise equipment were compared with the results of heart and breath sounds, respectively

To systematically evaluate the capability of the wearable sensor in clinical monitoring, we collected and analyzed two main types of cardiopulmonary acoustic signals. Specifically, Fig. 3a illustrates the physiological origin of heart sounds. The heart sound signal from an adult male was captured over a 1.5-s time window using the wearable stethoscope system, as shown in Fig. 3b. The signal clearly distinguishes between S1 (highlighted in yellow) and S2 (highlighted in green), providing key heart sound information such as the amplitude, period, time interval, and waveform shape of both S1 and S2. The spectrogram diagram in Fig. 3c illustrates the power intensity of different frequencies in the signal and their corresponding changes over time. The yellow regions at 0.25 and 0.5 s in the diagram represent S1 and S2, respectively.

Respiratory sounds are vibrations produced by the friction between the airflow and the inner walls of the trachea during respiration. Among these, bronchial respiratory sounds are generated when air enters the bronchus. These sounds are rough, loud, and high-pitched, with a strong and short duration during inhalation and a weak and long duration during exhalation. The frequency range of bronchial respiratory sounds typically spans from 10 Hz to 40 kHz, with pathological respiratory sounds often exhibiting higher frequencies. To accurately capture these broadband and subtle signals, sensors with high sensitivity, wide bandwidth, and flat frequency response are required. MEMS-based sensors provide a promising solution by enabling reliable detection of diverse physiological acoustic signals.

Meanwhile, Fig. 3d depicts the anatomical origin of respiratory sounds. Like the heart sound signal analysis, the sensor captures a 7-s time window respiratory sound signal, shown in Fig. 3e. The time-domain signal clearly reveals two complete inhalation and exhalation cycles, providing relevant respiratory sound information. Correspondingly, the spectrogram diagram in Fig. 3f displays the frequency and intensity distribution of the signal over time. As anticipated, the regular characteristics of the heart and lung sounds we observed are consistent with existing theoretical frameworks and research findings, further validating the accuracy of the underlying theory. In general, wearable stethoscopes excel in detecting human heart and lung sounds. They can capture sound signals with subtle changes that are often imperceptible to the human ear, enabling high-quality collection of heart and lung sound data. Moreover, the wearable stethoscope system is significantly smaller (34 times smaller in volume) and lighter (18 times lighter in weight) than traditional commercial stethoscopes, while offering advanced features like wireless transmission and charging. This makes continuous, hands-free real-time monitoring feasible. The system’s soft, flexible mechanical properties, coupled with its ability to perform time-synchronized operations via a wireless device network, set it apart as a highly functional and versatile medical tool.

To verify the reliability of the wearable stethoscope in cardiopulmonary measurements, we compared its performance with a standard commercial ECG device (outlined in gray) and a chest-motion monitoring system (outlined in blue). The heart sound test was conducted in the third intercostal space at the left edge of the sternum (outlined in red), while bronchial respiratory sounds were measured at the supraclavicular fossa (outlined in green). The placement positions corresponding to the four measurement methods are shown in the Supplementary Fig. S10. The results, shown in Fig. 3g, demonstrate a high degree of correlation between the wearable stethoscope and the standard devices. Over a 16-s time window, both heart testing methods recorded 20 cycles, with the S1 and S2 features of the heart sound closely matching the R and T peaks of the ECG data, confirming the expected pattern of ventricular contraction and relaxation. Additionally, the 16-s time window included four respiratory cycles, with chest motion closely aligning with the inhalation and exhalation phases of the bronchial respiratory sounds, further corroborating the relationship between chest movement and respiratory sounds. To assess the stethoscope’s applicability across different body types, the sensor was tested on six participants. Supplementary Fig. S11 presents the time-domain signal of heart sounds recorded over a 10-s window. Heart rate intervals, beat-to-beat intervals, and systolic and diastolic time intervals were calculated for the six participants and are shown in Table S4.

### Continuous monitoring of cardiac sounds in daily life

Auscultation of heart sounds is a critical method for evaluating cardiovascular diseases in clinical practice. The analysis of heart sounds and murmurs can provide valuable guidance for subsequent imaging tests and treatment decisions. For instance, the second heart sound is typically loudest in the aortic valve and pulmonary valve areas, helping to assess the function of the aortic valve, while the first heart sound is most prominent at the apex, often used to evaluate the function of the mitral and tricuspid valves. By analyzing the first and second heart sounds at the five main auscultation points—namely, the aortic valve area, pulmonary valve area, aortic valve second auscultation area, tricuspid valve area, and mitral valve area—key insights into heart valve function and hemodynamics can be gained. It is also important to note that certain characteristic murmurs can only be detected at specific auscultation points. For example, a systolic murmur caused by a ventricular septal defect is only audible in the tricuspid valve area, while a diastolic murmur caused by mitral stenosis is only detectable in the mitral valve area. Therefore, the ability of an auscultation system to record high-quality heart sound signals across all five auscultation points is crucial for accurate medical diagnosis.

**Fig. 4:**
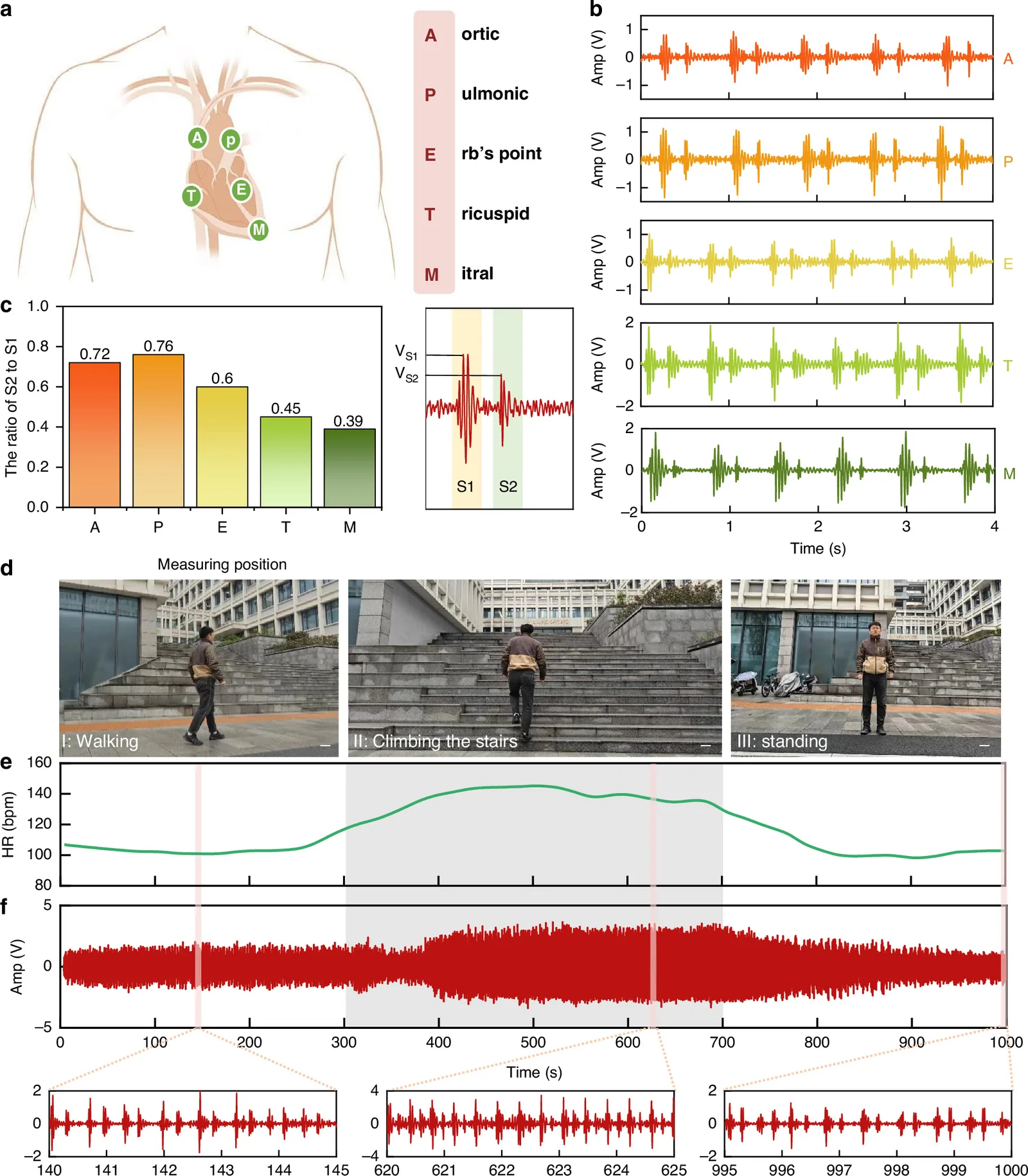
Clinical diagnosis of the five auscultation sites of testing and continuous monitoring during exercise. **a** The figure of five auscultation points on human skeleton. **b** The heart sound waveform measured on site in five auscultation points. **c** The ratio of S2 to S1 at the five auscultation sites. **d** Photographs showed participants wearing a wearable stethoscope performing three activities: (Ⅰ) walking, (Ⅱ) climbing stairs, and (Ⅲ) standing. Scale bar: 10 cm. **e**, **f** The participants’ **e** heart rate and **f** heart sounds were measured simultaneously in real time as they performed the activity

To further evaluate the potential of the wearable stethoscope system for medical diagnosis, we tested heart sound signals from the five key auscultation areas commonly used in clinical practice. Figure 4a shows the test positions for the aortic valve area, pulmonary valve area, second auscultation area of the aortic valve, tricuspid valve area, and mitral valve area. The heart sound signals collected by the wearable stethoscope system at these five locations are shown in Fig. 4b. For healthy participants, the S1 and S2 are critical parameters that can be routinely extracted and analyzed in these tests. Figure 4b shows that the intensity of the S1 in the tricuspid and mitral valve areas is significantly higher than in the other three auscultation points. Meanwhile, the S2 is particularly prominent in the aortic and pulmonary valve areas. To further analyze the characteristics of the five auscultation points, we calculated the amplitude ratio of S2 to S1 (Fig. 4c). In the aortic and pulmonary valve areas, the ratio of S2 to S1 was above 0.7, while in the tricuspid and mitral valve areas, the ratio was below 0.5, with S1 being the dominant sound. Additionally, in the second auscultation area of the aortic valve, both S1 and S2 were clearer, serving as a “compromise point” for listening to the sounds of both valves. The test results across the five auscultation points demonstrate the accuracy and reliability of the wearable auscultation system in capturing heart sound data, highlighting its potential for intelligent medical diagnosis.

To achieve high-quality, low-noise portable cardiopulmonary monitoring, we fabricated a rigid-encapsulated stethoscope and designed an experimental study to compare the superior performance of a flexible wearable stethoscope in maintaining close skin contact and minimizing motion artifacts. The heart sound signals were recorded simultaneously from two stethoscopes—rigid and flexible—during two activities, standing and walking, for 40 s. As shown in Fig. S12, the flexible wearable stethoscope clearly captured the peaks of S1 and S2 during walking, while the rigid device generated significant noise and motion artifacts. This illustrates why traditional stethoscopes are generally used only when the patient is at rest. The flexible wearable stethoscope we designed offers the advantages of conforming closely to the skin surface, buffering mechanical vibrations, and reducing friction noise generated by physical contact. As a result, the wearable flexible stethoscope significantly reduces sensitivity to noise from motion and physical contact, enabling clear detection of the first and second heart sound peaks (Fig. S12).

The wearable auscultation system is capable of continuously monitoring heart sound signals in real time during human motion. To evaluate its performance, we tested the system on participants who wore the sensors and engaged in various activities: normal walking for 300 s, climbing stairs for 400 s, and standing still for 300 s (Fig. 4d). During walking, the participants’ heart rate remained relatively stable (102–106 beats per minute). As they started climbing stairs, their heart rate gradually increased (116–145 beats per minute), while the heart sound signal strength remained stable at 3.6 V. In the standing position, the heart rate decreased from 130 beats per minute to 98 beats per minute over 200 s, and the heart sound signal strength dropped to 1.2 V. These results demonstrate that the wearable flexible auscultation system is a viable solution for health monitoring during physical activity.

### Quality monitoring and disease diagnosis during sleep

Sleep is a fundamental component of the human life cycle, with its quality and duration playing a crucial role in physical, mental, and social well-being^43,44^. Despite the increasing awareness of the importance of sleep quality for health, the number of individuals suffering from insufficient sleep continues to rise each year. The gold standard for sleep assessment is polysomnography (PSG), which uses multiple sensors to evaluate sleep quality and diagnose disorders. However, due to its high cost, complex system, and limited availability, many individuals with sleep disorders do not receive timely diagnoses^45^. Currently, Food and Drug Administration (FDA)-approved home tests for OSA involve complex systems with multiple components and are made from rigid materials that do not interface well with the skin^8,46^. We present a portable home sleep monitoring system that analyzes respiratory function by evaluating various types of respiratory sounds, thereby providing insights into the sleep quality or potential pathological conditions of participants. Unlike traditional polysomnography (PSG), which requires multiple wired sensors and bulky equipment, the developed wearable stethoscope offers a more convenient solution, allowing users to monitor their sleep in the comfort of their own home.

**Fig. 5:**
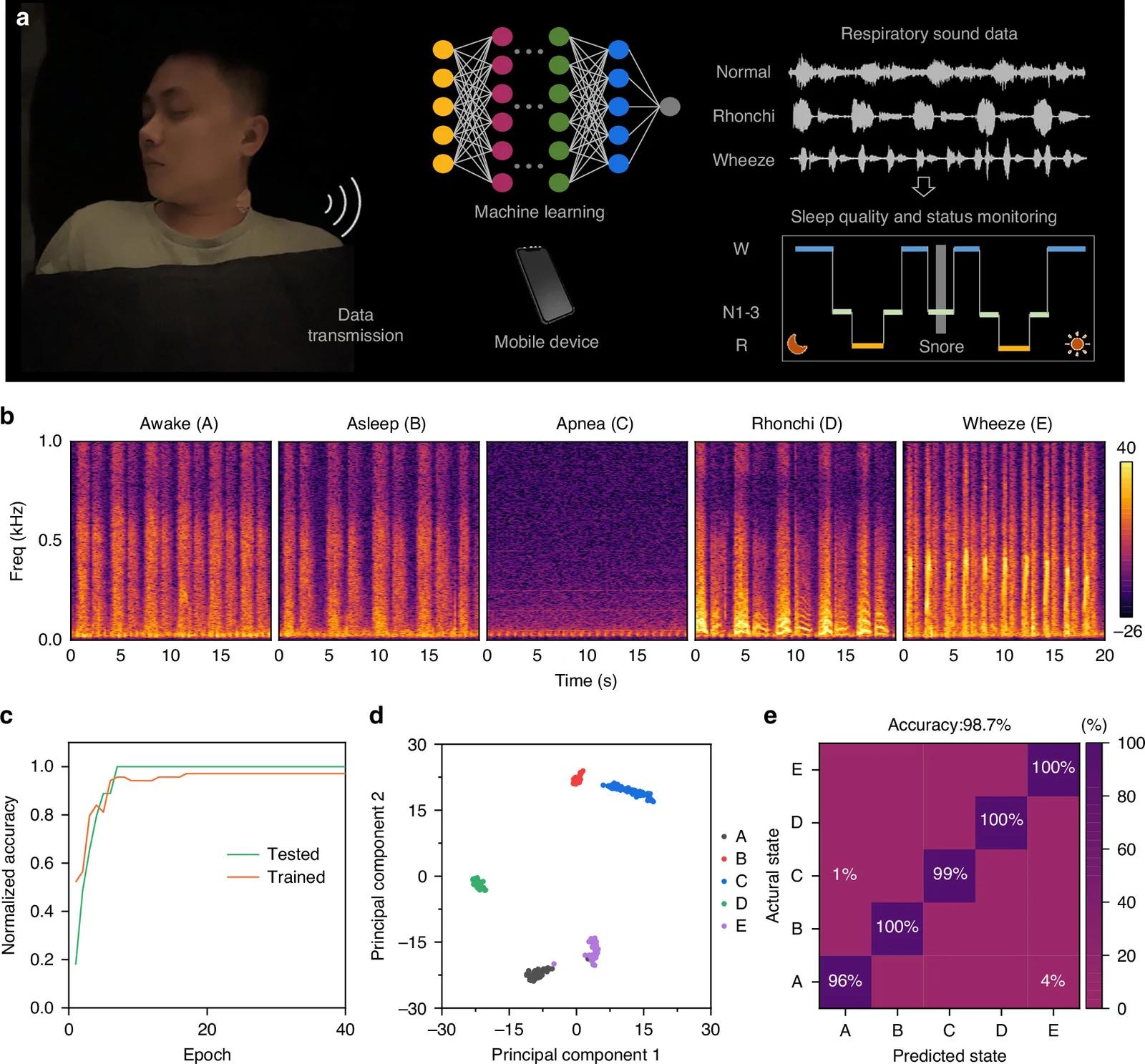
At-home sleep monitoring wearable stethoscope for assessing sleep quality and disorders. **a** Images of an at-home wireless sleep monitoring system. It is used to record breath sound signals in real time, and to classify sleep states and detect sleep disorders based on deep learning. **b** Spectrogram of five types of respiratory sounds in the 20-s time window, including wake state, sleep state, apnea, rhonchi and wheeze. **c** Normalized accuracy during 40-epoch iterations for the training and testing data. **d** Feature vector matrix after 40-epoch iterations processed by the T-SNE algorithm. **e** The confusion matrix of five types of respiratory sounds

The system begins with a flexible stethoscope placed on the supraclavicular fossa to collect data on bronchial respiratory sounds (Fig. 5a). The auscultation system captures five types of respiratory-related signals—awake, asleep, apnea, snoring, and wheezing—and converts these one-dimensional signals with distinctive features into spectrograms, creating two-dimensional vector data. Deep learning algorithms are then applied to a large dataset of experimental signals to classify them into valid categories with specific labels. Finally, the wearable stethoscope wirelessly transmits the collected data to a tablet or mobile phone via Bluetooth, enabling clinical-level sleep analysis. The respiratory sound signals corresponding to five sleep states were collected at a sampling rate of 10 kHz and converted into spectrograms using 20-s time windows (Fig. 5b). The spectrograms reveal significant differences in the respiratory frequency and signal strength among the awake, sleep, and apnea. Rhonchi is characterized by a low-frequency tone mixed with vibrational sounds, while wheeze exhibits higher frequencies, resembling sharp, piercing tones.

To process these signals, we employed a pretrained residual neural network as a foundation for transfer learning. The one-dimensional time-series signals captured by the wearable stethoscope were first converted into two-dimensional spectrogram images using the short-time Fourier transform and then analyzed through the residual neural network. The residual neural network employed in this study comprises 53 convolutional layers and one fully connected layer. During training, feature vectors were progressively refined as they passed through convolutional, pooling, and activation layers, reducing the dimensionality of the feature space. The extracted features were classified into specific targets by evaluating the consistency of the softmax function in the fully connected layer. The training model utilized data from five types of respiratory sounds as input sequences for classification. During preprocessing, the interval between two respiratory sound cycles was identified based on an amplitude threshold, and data collected within a 20-s time window were segmented into discrete respiratory sound samples. These time-domain signals were converted into spectrogram graphs and used as inputs for the network. The dataset was split into a training set (70%) and a validation set (30%) for model evaluation. After ten training iterations, the accuracy for all states in both the training and validation sets peaked (Fig. 5c), while the corresponding normalized loss approached zero (Fig. S13). The network’s classification results closely matched the actual categories, forming a confusion matrix (Fig. 5e). Principal component visualization using t-distributed stochastic neighbor embedding (T-SNE) demonstrated clear clustering of feature vectors in the machine learning space (Fig. 5d). The convolutional neural network achieved an overall prediction accuracy of 98.7% across the five studied states on both the training and validation sets. In summary, this wireless wearable auscultation system, integrated with machine learning technology, shows immense potential for applications in home healthcare, digital health monitoring, and quantitative disease diagnosis, significantly enhancing the scope of home-based sleep monitoring.

## Conclusion

In summary, we developed a flexible and wireless wearable stethoscope based on a 25-element circular AlN PMUT array. The packaged sensing system achieved a measured sensitivity of −167.5 dB, an operating frequency range of 10 Hz–10 kHz, and a frequency-response flatness of ±0.5 dB. Soft encapsulation and a conformal skin interface enabled stable acquisition of heart and respiratory sounds during stationary and dynamic activities. Human-subject measurements demonstrated heart-sound acquisition at five standard auscultation sites, temporal correspondence with reference ECG and chest-motion signals, and continuous heart-rate tracking during exercise. In a within-participant, session-separated proof-of-concept evaluation, the residual neural network classified five respiratory sound classes with an accuracy of 98.7%. Further studies using larger subject-independent datasets, clinically diagnosed participants, and systematic evaluations of battery endurance, wireless reliability, and long-term mechanical durability are required before clinical application.

## Methods

### Device finite element and design

The acoustic sensor was analyzed using the finite element method (FEM) with COMSOL Multiphysics 6.0 to study its vibration characteristics and frequency response under different piezoelectric materials and sensitive structures. The analysis considered square and circular sensor geometries, with structural dimensions specifically designed to unify the resonance frequency of all configurations at 1 MHz. In the FEM simulation, material properties were defined based on the selected piezoelectric materials, including PZT and AlN. Key parameters such as Young’s modulus, dielectric constant, and piezoelectric coefficients were adjusted within the model to accurately represent the physical behaviors of each material. This comprehensive approach ensured a detailed understanding of how material and structural variations influence the performance of the acoustic sensor.

### Device processing

The fabrication process of the MEMS sensor begins with the cleaning of the silicon-on-insulator (SOI) wafer (6N600-1-5N, Okmetic), which consists of a 5-μm-silicon device layer, a 1-μm-buried silicon oxide layer, and a 400-μm-silicon substrate. To enhance the surface quality for subsequent film deposition, a 100 nm AlN seed layer was grown using atomic layer deposition (ALD) (NLD-4000, Nano-master), significantly reducing surface roughness and improving the structural and morphological properties of both molybdenum (Mo) and c-axis-oriented AlN films. Next, physical vapor deposition (PVD) (Sigma, SPTS) was employed to sequentially deposit a stack of 0.3 μm Mo, 1 μm AlN, and another 0.3 μm Mo onto the AlN seed layer. The top Mo electrode pattern was defined in the subsequent patterning step through inductively coupled plasma (ICP) etching (GSEC200, NMC). This was followed by etching the AlN layer to create bottom electrode lead-out holes. Magnetron sputtering (MS150X-L, FHR) was then used to deposit a 200 nm Au layer, with metal leads and pads subsequently formed using the lift-off technique. Finally, deep reactive ion etching (DRIE) (Omega LPX Rapier, SPTS) was performed from the backside of the SOI wafer to release the cavities and form thin-film vibrational structures.

### Device characterization

The characterization of the device primarily focused on the micromachined MEMS acoustic sensor. Surface characterization was conducted using an optical microscope (MX63, Olympus), while the thickness and morphology of the piezoelectric films were analyzed with a scanning electron microscope (SU8600, Hitachi). To evaluate the electrical performance, a probe platform (EPS150RF, Cascade Microtech) and a vector network analyzer (E5080A, Keysight) were used to perform a frequency sweep in the range of 0.8 to 1.3 MHz, measuring the impedance and phase characteristics of the acoustic sensor. The growth quality of the AlN films was assessed using an X-ray diffraction instrument (Ultima IV, Rigaku), and the elemental composition of the functional layer was analyzed with an energy dispersive X-ray spectrometer (X-Max 50, Oxford). Finally, the MEMS sensor was electrically connected to an external FPCB using a wire bonding machine (WB-91D, BEE), with a gold wire of 25 μm in diameter. This comprehensive characterization ensured a detailed understanding of the sensor’s structural, material, and performance properties.

### Signal processing, transmission, and power circuits

The amplified and filtered analog electrical signal is digitized by the analog-to-digital converter (ADC) module (ADS78223). Under the control of the microcontroller unit (MCU) main control module (ESP32), the digitized data is wirelessly transmitted to a personal computer (PC) host via Bluetooth (Supplementary Fig. S2).

### Device flexible packaging

The schematic diagram of the wearable stethoscope flexible package preparation process is shown in Supplementary Fig. S14. The process is as follows: mix the A and B components of silicone (Ecoflex-0030) in a 1:1 ratio at room temperature; place the mixed silicone in a vacuum box for degassing for 5 min to remove air bubbles; pour the degassed silicone mixture into a pre-designed mold, ensuring the entire flexible circuit board of the sensor is covered with silicone; place the mold back into the vacuum box and degas for an additional 5 min to eliminate any remaining bubbles; allow the silicone mixture to stand at room temperature for 4 h to solidify, resulting in the MEMS skin attachment layer. This process ensures a reliable and flexible packaging suitable for sensor applications.

### Collection of sound data

The wearable stethoscope was installed in the third intercostal space on the left side of the sternum to detect heart sounds. The stethoscope was installed in the supraclavicular fossa to detect bronchial respiratory sounds. The recording duration was up to 1000 s. In addition, in the sports scenario, the test subjects were asked to do different activities, including walking, climbing stairs, and standing.

### Data acquisition and analysis

The original ADC output of the sensor is sampled through a data acquisition device, and the collected one-dimensional time-domain signal is analyzed using MATLAB. The signal processing procedure includes applying a second-order bandpass filter to isolate the desired frequency components, followed by transforming the filtered signal into a two-dimensional spectrogram representation using a short-time Fourier transform.

### Machine learning algorithms for classification and prediction

We used a pretrained residual neural network as the foundation for transfer learning. The network architecture consists of 53 convolutional layers and one fully connected layer. In the training model, the processing vector iteratively passes through convolution, pooling, and activation layers to achieve dimensionality reduction of the feature vector. The training parameters of the network are detailed in Supplementary Table S5. All measurement data from one participant were labeled with distinct feature types and normalized to unify data dimensions. Of this data, 70% of the continuous measurements from a single session were used as training data, while the remaining 30% served as validation data. Test data from a different session were used exclusively for evaluation. During training, feature vectors clustered using the T-SNE algorithm provided a visual representation of the principal components’ evolution within the machine learning space, aiding in the interpretation and optimization of the model.

## Supplementary information

Supporting Information

## References

[1] Townsend, N., Nichols, M., Scarborough, P. & Rayner, M. Cardiovascular disease in Europe — epidemiological update 2015. *Eur. Heart J.* **36**, 2696–2705 10.1093/eurheartj/ehv428 (2015). 26306399

[2] Benjafield, A. V. et al. Estimation of the global prevalence and burden of obstructive sleep apnoea: a literature-based analysis. *Lancet Respir. Med.* **7**, 687–698 10.1016/s2213-2600(19)30198-5 (2019). PMC7007763 31300334

[3] Strollo, P. J. et al. Upper-airway stimulation for obstructive sleep apnea. *N. Engl. J. Med.* **370**, 139–149 10.1056/NEJMoa1308659 (2014). 24401051

[4] Kluytmans-van den Bergh, M. F. Q. et al. Prevalence and clinical presentation of health care workers with symptoms of coronavirus disease 2019 in 2 Dutch hospitals during an early phase of the pandemic. *JAMA Netw. Open* **3**, e209673–e209673 10.1001/jamanetworkopen.2020.9673 (2020). PMC7243090 32437576

[5] Virani, S. S. et al. Heart disease and stroke statistics—2020 update: a report from the American Heart Association. *Circulation* **141**, e146–e603, 10.1161/cir.0000000000000757 (2020). 31992061

[6] Yeghiazarians, Y. et al. Obstructive sleep apnea and cardiovascular disease: a scientific statement from the American Heart Association. *Circulation* **144**, e56–e67 10.1161/cir.0000000000000988 (2021). 34148375

[7] Jeong, H., Rogers, J. A. & Xu, S. Continuous on-body sensing for the COVID-19 pandemic: gaps and opportunities. *Sci. Adv.* **6**, eabd4794 10.1126/sciadv.abd4794 (2020). PMC7467694 32917604

[8] Watson, N. F., Rosen, I. M. & Chervin, R. D. The past is prologue: the future of sleep medicine. *J. Clin. Sleep Med.* **13**, 127–135 10.5664/jcsm.6406 (2017). PMC5181604 27998380

[9] Sullivan, F. *Hidden Health Crisis Costing America Billions: Underdiagnosing and Undertreating Obstructive Sleep Apnea Draining Healthcare System* (American Academy of Sleep Medicine, 2016).

[10] Huang, L. et al. Cardiac involvement in patients recovered from COVID-2019 identified using magnetic resonance imaging. *JACC Cardiovasc. Imaging* **13**, 2330–2339 10.1016/j.jcmg.2020.05.004 (2020). PMC7214335 32763118

[11] Collier, P., Phelan, D. & Klein, A. A Test in context: myocardial strain measured by speckle-tracking echocardiography. *J. Am. Coll. Cardiol.* **69**, 1043–1056 10.1016/j.jacc.2016.12.012 (2017). 28231932

[12] Caballeros Lam, M., de la Fuente Villena, A., Hernández Hernández, A., García de Yébenes, M. & Bastarrika Alemañ, G. Cardiac magnetic resonance characterization of COVID-19 myocarditis. *Rev. Esp. Cardiol.* **73**, 863–864 10.1016/j.rec.2020.06.018 (2020). PMC7334914 32694081

[13] Voigt, J. U. & Cvijic, M. 2-and 3-dimensional myocardial strain in cardiac health and disease. *JACC Cardiovasc. Imag.* **12**, 1849–1863 10.1016/j.jcmg.2019.01.044 (2019). 31488253

[14] Hahn, R. T. et al. Imaging assessment of tricuspid regurgitation severity. *JACC: Cardiovasc. Imaging* **12**, 469–490 10.1016/j.jcmg.2018.07.033 (2019). 30947907

[15] Harmon, S. A. et al. Artificial intelligence for the detection of COVID-19 pneumonia on chest CT using multinational datasets. *Nat. Commun.* **11**, 4080 10.1038/s41467-020-17971-2 (2020). PMC7429815 32796848

[16] Pan, F. et al. Time course of lung changes at chest CT during recovery from coronavirus disease 2019 (COVID-19). *Radiology* **295**, 715–721 10.1148/radiol.2020200370 (2020). PMC7233367 32053470

[17] Groves, A. M. et al. Idiopathic pulmonary fibrosis and diffuse parenchymal lung disease: implications from initial experience with18F-FDG PET/CT. *J. Nucl. Med.* **50**, 538–545, 10.2967/jnumed.108.057901 (2009). 19289428

[18] Han, L. et al. Stretchable piezoelectret electronic stethoscope for phonocardiography and lung sound detection in motion and noise conditions. *Appl. Mater. Today* **36**, 102077 10.1016/j.apmt.2024.102077 (2024).

[19] Zhang, B. B. et al. A three-dimensional liquid diode for soft, integrated permeable electronics. *Nature* 84–92 10.1038/s41586-024-07161-1 (2024). 38538792

[20] Hui, X. et al. Acoustically enhanced triboelectric stethoscope for ultrasensitive cardiac sounds sensing and disease diagnosis. *Adv Mater* **36**, e2401508 10.1002/adma.202401508 (2024). 38747492

[21] Tseng, Y. L., Ko, P. Y. & Jaw, F. S. Detection of the third and fourth heart sounds using Hilbert-Huang transform. *Biomed. Eng. Online* **11**, 1–13 (2012). 10.1186/1475-925X-11-8 PMC3305384 22332995

[22] Muthusamy, P. D., Sundaraj, K. & Abd Manap, N. Computerized acoustical techniques for respiratory flow-sound analysis: a systematic review. *Artif. Intell. Rev.* **53**, 3501–3574 10.1007/s10462-019-09769-6 (2019).

[23] Fu, J. et al. Estimation of respiratory nasal pressure and flow rate signals using different respiratory sound features. *IRBM* **43**, 694–704 10.1016/j.irbm.2021.12.002 (2022).

[24] Lee, S. H. et al. Fully portable continuous real-time auscultation with a soft wearable stethoscope designed for automated disease diagnosis. *Sci. Adv.* **8**, eabo5867 10.1126/sciadv.abo5867 (2022). PMC9132462 35613271

[25] Qiu, C. et al. Wearable stethoscope for lung disease diagnosis. *Sens. Diagn.* **3**, 281–286 10.1039/d3sd00283g (2024).

[26] Kim, Y. et al. Respiratory sound classification for crackles, wheezes, and rhonchi in the clinical field using deep learning. *Sci. Rep.* **11**, 1–11 10.1038/s41598-021-96724-7 (2021). PMC8387488 34433880

[27] Lee, S. H., Kim, Y.-S. & Yeo, W.-H. Advances in microsensors and wearable bioelectronics for digital stethoscopes in health monitoring and disease diagnosis. *Adv. Healthc. Mater.* **10** 10.1002/adhm.202101400 (2021). 34486237

[28] Wen, D.-L. et al. High-performance hybrid nanogenerator for self-powered wireless multi-sensing microsystems. *Microsyst. Nanoeng*. **9** 10.1038/s41378-023-00563-7 (2023). PMC10359314 37484504

[29] Gupta, P. et al. Precision wearable accelerometer contact microphones for longitudinal monitoring of mechano-acoustic cardiopulmonary signals. *Npj Digit. Med.* **3**, 19, 10.1038/s41746-020-0225-7 (2020). PMC7015926 32128449

[30] Kang, Y. J. et al. Soft skin-interfaced mechano-acoustic sensors for real-time monitoring and patient feedback on respiratory and swallowing biomechanics. *Npj Digit. Med.* **5**, 147 10.1038/s41746-022-00691-w (2022). PMC9485153 36123384

[31] Kim, Y. S. et al. All-in-one, wireless, stretchable hybrid electronics for smart, connected, and ambulatory physiological monitoring. *Adv. Sci.* **6**, 1900939 10.1002/advs.201900939 (2019). PMC6724359 31508289

[32] Kölle, K., Aftab, M. F., Andersson, L. E., Fougner, A. L. & Stavdahl, Ø. Data driven filtering of bowel sounds using multivariate empirical mode decomposition. *Biomed. Eng. Online* **18**, 1–20 10.1186/s12938-019-0646-1 (2019). PMC6425713 30894187

[33] Chung, H. U. et al. Skin-interfaced biosensors for advanced wireless physiological monitoring in neonatal and pediatric intensive-care units. *Nat. Med.* **26**, 418–429 10.1038/s41591-020-0792-9 (2020). PMC7315772 32161411

[34] Liu, T. et al. Emerging wearable acoustic sensing technologies. *Adv. Sci.* **12**, e2408653 10.1002/advs.202408653 (2025). PMC11809411 39749384

[35] Jia, L. C. et al. Piezoelectric micromachined ultrasonic transducer array-based electronic stethoscope for internet of medical things. *IEEE Internet Things* **9**, 9766–9774 (2022).

[36] Chen, H. B. et al. A two-stage amplified PZT sensor for monitoring lung and heart sounds in discharged pneumonia patients. *Microsyst. Nanoeng.* **7**, 55 10.1038/s41378-021-00274-x (2021). PMC8433369 34567768

[37] Cotur, Y. et al. Stretchable composite acoustic transducer for wearable monitoring of vital signs. *Adv. Funct. Mater.* **30**, 1910288 10.1002/adfm.201910288 (2020). PMC7116191 33071715

[38] Nayeem, M. O. G. et al. All-nanofiber-based, ultrasensitive, gas-permeable mechanoacoustic sensors for continuous long-term heart monitoring. *Proc. Natl. Acad. Sci. USA* **117**, 7063–7070 10.1073/pnas.1920911117 (2020). PMC7132136 32188781

[39] Li, Y. H., Huang, Y. & Zhao, N. Low-intensity sensitive and high stability flexible heart sound sensor enabled by hybrid near-field/far-field electrospinning. *Adv. Funct. Mater.* **33** 2300666. 10.1002/adfm.202300666 (2023).

[40] Liu, T. et al. Airborne rangefinding with pMUTs array using differential structure. *IEEE Sens. J.* **23**, 22240–22247 10.1109/Jsen.2023.3298671 (2023).

[41] Liu, T. et al. Intelligent monitoring system for pipeline status based on MEMS acoustic emission sensors. *Microsyst. Nanoeng.* **12**, 260, 10.1038/s41378-026-01367-1 (2026). PMC13351072 42425935

[42] Liu, T. et al. Machine learning-assisted wearable sensing systems for speech recognition and interaction. *Nat. Commun.* **16** 10.1038/s41467-025-57629-5 (2025). PMC11894117 40064879

[43] Gulia, K. K. & Kumar, V. M. Importance of sleep for health and wellbeing amidst COVID-19 pandemic. *Sleep Vigil.* **4**, 49–50 10.1007/s41782-020-00087-4 (2020). PMC7197235 32368715

[44] Buysse, D. J. Sleep health: can we define it? Does it matter? *Sleep* **37**, 9–17 10.5665/sleep.3298 (2014). PMC3902880 24470692

[45] Ibáñez, V., Silva, J., Navarro, E. & Cauli, O. Sleep assessment devices: types, market analysis, and a critical view on accuracy and validation. *Expert Rev. Med. Dev.* **16**, 1041–1052 10.1080/17434440.2019.1693890 (2019). 31774330

[46] Punjabi, N. M., Patil, S., Crainiceanu, C. & Aurora, R. N. Variability and misclassification of sleep apnea severity based on multi-night testing. *Chest* **158**, 365–373 10.1016/j.chest.2020.01.039 (2020). PMC7339240 32081650

