# Supplementary material for: Broadband wearable acoustic sensing for continuous cardiopulmonary monitoring
Source: Microsyst Nanoeng. 2026 Sep 29;12:334. doi: 10.1038/s41378-026-01460-5 (PMC13619561; doi:10.1038/s41378-026-01460-5)
Supplement: Supplementary file 1 [file 41378_2026_1460_MOESM1_ESM.doc]

Supporting Information

**Broadband Wearable Acoustic Sensing for Continuous Cardiopulmonary Monitoring**

Pengfan Wu^1,4,†^, Tao Liu^2,†,*^, Dongxiao Li^1,†^, Hanjie Dou^1,4^, Jiaqian Yang^1,4^, Hongliang Wang^2^, Hengyu Guo^3^, Xiaojing Mu^1,4,^ *

**Author Information**

Affiliations

**1. Key Laboratory of Optoelectronic Technology & Systems Ministry of Education, International R & D center of Micro-nano Systems and New Materials Technology, Chongqing University, Chongqing, 400044, P.R. China**

**2. Science and Technology on Electronic Test and Measurement Laboratory, State key Laboratory of Extreme Environment Optoelectronic Dynamic Measurement Technology and Instrument, North University of China, Taiyuan, 030051, P.R. China**

**3. College of Physics, Chongqing University, Chongqing 400044, China**

**4. Jialingjiang Laboratory, Chongqing 401331, China.**

^†^These authors contributed equally to this work.

### Corresponding author

E-mail: liutao@nuc.edu.cn; [mxjacj@cqu.edu.cn](mailto:mxjacj@cqu.edu.cn)

**Supplementary Figure S1. Schematic diagram of component layout of flexible printed circuit board (FPCB). a** Distribution diagram of components and functional modules on the front of FPCB. **b** Topography diagram of FPCB base. **c** Distribution diagram of components and functional modules on the back of FPCB.

**Supplementary Figure S2. Schematic diagrams of the integrated hardware circuitry for the MEMS sensor. a** JFET-based low-noise preamplifier circuit. **b** Sallen-Key sixth-order high-pass and low-pass filter circuits for signal conditioning. **c** Analog-to-digital conversion (ADC) circuit. **d** Lithium battery charge management circuit. **e** Microcontroller unit (MCU) module circuit. **f** Boost and inverting power supply circuit.

The formula for calculating the signal-to-noise ratio is as follows,

$SNR=10\lg\left[\frac{\overline{P_{S}}}{\overline{P_{N}}}\right]=10\lg(\frac{\overline{A_{s}}}{\overline{A_{N}}})^{2}$ 1

Where $\overline{P_{S}}$ and $\overline{P_{N}}$ are the average power of the output signal and the average power of the sensor background noise, $\overline{A_{S}}$ is the average value of the voltage amplitude of the MEMS sensor detection signal, and $\overline{A_{N}}$ is the average value of the noise signal voltage amplitude. Through the voice test of participants, the output noise and voice signal of the flexible stethoscope are shown in the figure below, and the SNR is finally calculated to be 40.09 dB.

**Supplementary Figure S3. The signal-to-noise ratio test of the wearable auscultation system. a** The average voltage of the sound signal obtained by the participants through the wearable auscultation system test is 2 V. **b** The average output noise signal voltage of the wearable auscultation system is 0.019V.

To ensure the effective electromechanical conversion of the ultrasonic transducer, the neutral axis needs to be located in the silicon device layer. The properties of each layer of materials that make up the membrane structure have a great influence on the position of the neutral axis. The position of the neutral axis is expressed as follows:

2

where *H_np_* is the location of the neutral axis, *Y_n_* is the Young's modulus, *υ_n_* is the Poisson's ratio, *t_n_* is the thickness of the n layer, and *h_n_* is the z-axis position of the upper part of the *n* layer.

3

4

5

where *a* is the radius, *D* is the stiffness of the plate, *μ* is the mass per area of the plate, and *ρ_n_* is the density of the different materials. Considering the co-design of sensor sensitivity and flatness, the resonant frequency is finally determined to be 1 MHz.

**Supplementary Figure S4. Working principle diagram of piezoelectric micromachined ultrasonic transducer (PMUT).** Here, the cavity causes the sensor to generate a bending moment about the neutral axis in the stress gradient along the thickness of the film in the area covered by the electrode, causing the film to vibrate.

**Supplementary Figure S5. Analysis of the relationship between transformation ratio and the ratio of top electrode radius to cavity ratio by finite element method (FEM) model.**

**Supplementary Figure S6 Finite element simulation of frequency response curve of MEMS acoustic sensor. a** Frequency response curve of MEMS sensor in 0 - 1.2 MHz band. **b** Local amplification frequency response curve of MEMS sensor in 0 – 10 kHz band. The resonant frequency of our sensor in the air domain is 1 MHz, which can ensure the sensitivity of the sensor and also ensure its flatness in the wide band.

**Supplementary Figure S7. The fabrication process of the acoustic sensor.** **a** The processing process of the MEMS sensor starts with the cleaning of the silicon on insulator (SOI). The SOI wafer consists of a 5 μm silicon device layer, a 1 μm buried silicon oxide layer, and a 400 μm silicon substrate. **b** AlN seed layer of about 100 nm was grown through atomic layer deposition (ALD) (NLD-4000, Nano-master) to reduce the surface roughness of subsequent film deposition, thereby improving the structure and morphology of Mo and c-axis oriented AlN. Physical vapor phase Deposition (PVD) was then used to sequentially deposit 0.3 μm Mo, 1 μm AlN, and 0.3 μm Mo on the AlN seed layer. (**c**-**e**) In the subsequent patterning step, inductively coupled plasma etching (ICP) was used to form the top Mo electrode pattern, AlN piezoelectric layer, Mo bottom electrode. **f** A 300 nm thick silicon oxide was grown on the front side of the wafer by PECVD (SYSTEM 100), and 300nm of the silicon oxide was etched by ICP (STS AOE) to expose the top electrode Mo and the bottom electrode Mo. **g** 200 nm Au was deposited by magnetron sputtering and metal leads and pads were formed by the lift-off method. **h** Finally, deep reactive ion etching (DRIE) was performed from the back of the SOI to release cavities and form thin film vibration structures.

**Supplementary Figure S8.** Grooves about 20 μm wide × 20 μm long × 10 μm deep were opened on the surface of AlN thin films using a focused ion beam (FIB, Scios dual-beam scanning electron microscopy /FIB system). The scandium concentration on AlN cross section was measured by energy dispersive X-ray spectrometer at 15 keV beam energy. EDX scanning data is shown in the figure. The results show that the scandium concentration remains consistent throughout the film thickness. **a** corresponding EDX mappings of four key elements for observing Si and AlN layers. Overall EDX topography of **b** the film and **c** individual element.

**Supplementary Figure S9. The crystal structure of AlN was studied by X-ray diffraction (XRD).**

**Supplementary Figure S10. Experimental setup and physical attachment positions for cardiopulmonary monitoring.** (Left panel) Configuration for heart sound measurement. The proposed wearable sensor is attached to the third intercostal space at the left edge of the sternum, alongside the placement of a standard commercial ECG device for synchronous comparison. (Right panel) Configuration for breath sound measurement. The proposed sensor is placed at the supraclavicular fossa to capture bronchial respiratory sounds, paired with a commercial chest motion monitoring system (respiratory band).

**Supplementary Figure S11 The stethoscope was tested on six participants of different body types.**

**Supplementary Figure S12. Comparison of stethoscope performance in overcoming motion artifacts.** Time series of the rigid stethoscope **a** versus the flexible stethoscope **b** mounted on the chest. The subjects performed different activities including standing and walking while the sounds were recorded. A partial enlargement of the heart sounds collected by **c** the rigid and **d** flexible stethoscopes within a 2-s time window.

**Supplementary Figure S13. Normalized loss of training and test data during 40 epoch iterations.**

**Supplementary Figure S14. The schematic diagram of the flexible package preparation process.**

**Supplementary Figure S15. Current testing of device**

**Table S1** Performance comparison of digital stethoscopes.

| **Ref** | **Size**  **(mm)** | **Weight**  **(g)** | **Signal Bandwidth** | **SNR**  **(dB)** | **Data**  **Communication** | **Collected Data** |
| --- | --- | --- | --- | --- | --- | --- |
| This work | 56×25×5 | 6.8 | 10Hz-10kHz | 40.09 | Bluetooth | Heart sound,  Lung sound |
| 3M^TM^ Littmann CORE | 175×45×30 | 127.3 | 20Hz-40kHz | 35 | Bluetooth | Body sound |
| Lee et al. ^1^ | / | / | 20Hz-2kHz | 14.8 | Bluetooth | Heart sound,  Lung sound |
| Rao et al. ^2^ | / | / | 50Hz-1kHz | 10 | Bluetooth | Lung sound |
| Wang et al. ^3^ | 41.9×30.2×7.8 | 6.5 | 50Hz-2kHz | 7 | Bluetooth | Bowel sound |
| Chowdhury et al. ^4^ | / | / | 20Hz-2kHz | 10 | Bluetooth | Heart sound |
| Shimoda et al. ^5^ | / | / | 50Hz-12.5kHz | / | Wire communication | Lung sound |
| Islam et al. ^6^ | / | / | 70Hz-8kHz | / | Wire communication | Lung sound |

**Table S2** Parameter comparison of PMUT stacking materials.

|  | **Mo** | **AlN** | **Si** | **SiO_2_** |
| --- | --- | --- | --- | --- |
| Young modulus  E (Gpa) | 312 | 310 | 170 | 60 |
| Density  ρ (kg/m^3^) | 10200 | 3260 | 2329 | 2200 |
| Poisson's ratio  σ | 0.3 | 0.22 | 0.28 | 0.17 |

**Table S3** Comparison of parameters of different piezoelectric materials.

| **Comparison** | **PZT** | **ZnO** | **AlN** |
| --- | --- | --- | --- |
| Density [kg/m^3^] | ~7500 | ~5680 | -3260 |
| Longitudinal  Velocity [m/s] | ~4600 | ~6350 | ~11300 |
| Transverse  velocity [m/s] | ~2000 | ~2720 | ~6000 |
| Theral Conductivlty [W/(m·K)] | -1.8 | 60 | 280 |
| TCF [ppm/°G] | NA | -120 | -50 |
| e_31_ [C/m^2^] | -10 | -1 | -1,05 |
| ε_33_ | 1000 | 10.9 | 10..5 |
| Rx Sensitivity ∝d_31_/ε_33_ | -0.01 | -0.092 | -0.1 |

**Table S4** Heart rate status parameters of 6 participants.

| **Participant** | **Gender** | **Body mass**  **index** | **Heart rate interval**  **(bpm)** | **Interbeat interval (s)** | **Systolic time interval (s)** | **Diastolic time interval (s)** |
| --- | --- | --- | --- | --- | --- | --- |
| A | M | 19 | 57 | 1.05 | 0.36 | 0.69 |
| B | M | 24 | 73 | 0.88 | 0.31 | 0.57 |
| C | F | 26 | 81 | 0.72 | 0.25 | 0.47 |
| D | F | 22 | 80 | 0.74 | 0.28 | 0.46 |
| E | M | 32 | 81 | 0.74 | 0.31 | 0.43 |
| F | M | 22 | 71 | 0.85 | 0.3 | 0.55 |

**Table S5** Essential parameters used in model training.

| **ResNet18** | |
| --- | --- |
| Momentum | 0.9 |
| InitialLearnRate | 0.001 |
| LearnRateSchedule | None |
| LearnRateDropFactor | 0.1 |
| LearnRateDropPeriod | 10 |
| L2Regularization | 0.0001 |
| GradientThresholdMethod | 12norm |
| GradientThreshold | inf |
| MaxEpochs | 30 |
| MiniBatchSize | 512 |
| Verbose | 1 |
| VerboseFrequency | 50 |

**References**

1	Lee, S. H. *et al.* Fully portable continuous real-time auscultation with a soft wearable stethoscope designed for automated disease diagnosis. *Sci. Adv.* **8**, eabo5867, doi:ARTN eabo586710.1126/sciadv.abo5867 (2022).

2	Rao, A., Ruiz, J., Bao, C. & Roy, S. Tabla: A Proof-of-Concept Auscultatory Percussion Device for Low-Cost Pneumonia Detection. *Sensors-Basel* **18**, doi:10.3390/s18082689 (2018).

3	Wang, F. *et al.* A flexible skin-mounted wireless acoustic device for bowel sounds monitoring and evaluation. *Science China Information Sciences* **62**, doi:10.1007/s11432-019-9906-1 (2019).

4	Chowdhury, M. E. H. *et al.* Real-Time Smart-Digital Stethoscope System for Heart Diseases Monitoring. *Sensors-Basel* **19**, doi:10.3390/s19122781 (2019).

5	Shimoda, T. *et al.* Lung sound analysis helps localize airway inflammation in patients with bronchial asthma. *Journal of Asthma and Allergy* **Volume10**, 99-108, doi:10.2147/jaa.S125938 (2017).

6	Islam, M. A., Bandyopadhyaya, I., Bhattacharyya, P. & Saha, G. Multichannel lung sound analysis for asthma detection. *Computer Methods and Programs in Biomedicine* **159**, 111-123, doi:10.1016/j.cmpb.2018.03.002 (2018).
